# Integrative analysis identifies lincRNAs up- and downstream of neuroblastoma driver genes

**DOI:** 10.1038/s41598-019-42107-y

**Published:** 2019-04-05

**Authors:** Dries Rombaut, Hua-Sheng Chiu, Bieke Decaesteker, Celine Everaert, Nurten Yigit, Agathe Peltier, Isabelle Janoueix-Lerosey, Christoph Bartenhagen, Matthias Fischer, Stephen Roberts, Nicky D’Haene, Katleen De Preter, Frank Speleman, Geertrui Denecker, Pavel Sumazin, Jo Vandesompele, Steve Lefever, Pieter Mestdagh

**Affiliations:** 1 Center for Medical Genetics, Ghent University, Ghent, 9000 Belgium; 2 Cancer Research Institute Ghent (CRIG), Ghent, 9000 Belgium; 3 Texas Children’s Cancer Center, Baylor College of Medicine, Houston, TX 77030 USA; 4 Institut Curie, PSL Research University, Inserm U830, Equipe Labellisée contre le Cancer, F-75005 Paris, France; 5 SIREDO: Care, Innovation and Research for Children, Adolescents and Young Adults with Cancer, Institut Curie, F-75005 Paris, France; 6 Center for Molecular Medicine Cologne (CMMC), University of Cologne, 50931 Cologne, Germany; 7 Department of Experimental Pediatric Oncology, University Children’s Hospital of Cologne, Medical Faculty, University of Cologne, 50937 Cologne, Germany; 8 Department of Pediatrics, Memorial Sloan Kettering Cancer Center, New York, NY USA; 9 Hôpital Erasme, Cliniques Universitaires de Bruxelles, Bruxelles, 1070 Belgium

**Keywords:** Paediatric cancer, Cancer genomics

## Abstract

Long intergenic non-coding RNAs (lincRNAs) are emerging as integral components of signaling pathways in various cancer types. In neuroblastoma, only a handful of lincRNAs are known as upstream regulators or downstream effectors of oncogenes. Here, we exploit RNA sequencing data of primary neuroblastoma tumors, neuroblast precursor cells, neuroblastoma cell lines and various cellular perturbation model systems to define the neuroblastoma lincRNome and map lincRNAs up- and downstream of neuroblastoma driver genes *MYCN*, *ALK* and *PHOX2B*. Each of these driver genes controls the expression of a particular subset of lincRNAs, several of which are associated with poor survival and are differentially expressed in neuroblastoma tumors compared to neuroblasts. By integrating RNA sequencing data from both primary tumor tissue and cancer cell lines, we demonstrate that several of these lincRNAs are expressed in stromal cells. Deconvolution of primary tumor gene expression data revealed a strong association between stromal cell composition and driver gene status, resulting in differential expression of these lincRNAs. We also explored lincRNAs that putatively act upstream of neuroblastoma driver genes, either as presumed modulators of driver gene activity, or as modulators of effectors regulating driver gene expression. This analysis revealed strong associations between the neuroblastoma lincRNAs *MIAT* and *MEG3* and *MYCN* and *PHOX2B* activity or expression. Together, our results provide a comprehensive catalogue of the neuroblastoma lincRNome, highlighting lincRNAs up- and downstream of key neuroblastoma driver genes. This catalogue forms a solid basis for further functional validation of candidate neuroblastoma lincRNAs.

## Introduction

During the past decade, detailed analysis of the human transcriptome revealed thousands of RNA molecules with no obvious coding potential^1–5^. These so-called long non-coding RNAs (lncRNAs) are poorly conserved at the sequence level and have a lower but more cell-type specific expression profile compared to protein coding mRNAs^1,6,7^. Based on functional studies of selected lncRNAs, it has become clear that they can act as important modulators of various processes in the cell, including chromatin conformation, transcription, splicing and post-transcriptional regulation^8–10^. Their capacity to interact with several bio-molecules in the cell (i.e. RNA, DNA and proteins) provides them with a plethora of mechanisms to exert their functions. Not surprisingly, deregulated expression of lncRNAs may cause human diseases including cancer^8,11–14^. At present, dozens of lncRNAs are known to function up- or downstream of cancer drivers or key signaling pathways. Notable examples are *TP53* pathway tumor suppressor lncRNAs *PANDAR* and *lincRNA-p21*^15,16^ and oncogenic lncRNAs *CCAT2* and *MINCR* as respective activator or effector of *MYC*^17^. Systematic analysis of RNA-sequencing data across various adult tumor types further demonstrated that mutations in oncogenes and tumor suppressor genes can deregulate lncRNA expression^18^. Knockdown of lncRNAs driving oncogenic signaling can result in a therapeutic response *in vitro* and *in vivo*^17,19,20^.

Neuroblastoma (NB) is one of the most enigmatic tumors, with clinical behavior ranging from spontaneous regression to metastatic disease refractory to aggressive multimodal therapy^21^. Tumors arise from neural crest-derived progenitor cells through deregulation of signaling pathways governing sympathetic nervous system development and differentiation. Despite the identification of predisposing mutations (*PHOX2B*^22^), somatic mutations (*ALK*^23,24^*, ATRX*^25^), amplifications (*MYCN*^26^) and translocations (TERT^27^) affecting several protein-coding genes, little progress was made in improving overall survival in the last decade. Unravelling the lncRNA components involved in these signaling pathways could help us better understand how these networks are wired and may reveal novel regulatory mechanisms and, ultimately, therapeutic strategies. While only a handful of NB-associated lncRNAs have been identified today, for some, a putative therapeutic targeting potential has been demonstrated. *NBAT1*, a lncRNA on chromosome 6, possesses tumor suppressor functions, inhibiting NB proliferation and invasion and promoting differentiation^28^. Other examples of deregulated lncRNAs in NB are *ncRAN*, located on chromosome 17q and associated with poor prognosis, MYCN target gene *linc00467* that represses *DKK1* leading to increased NB cell survival and *lncUSMycN*, located on 2p and shown to regulate *MYCN* expression post-transcriptionally^29–31^.

**Figure 1:**
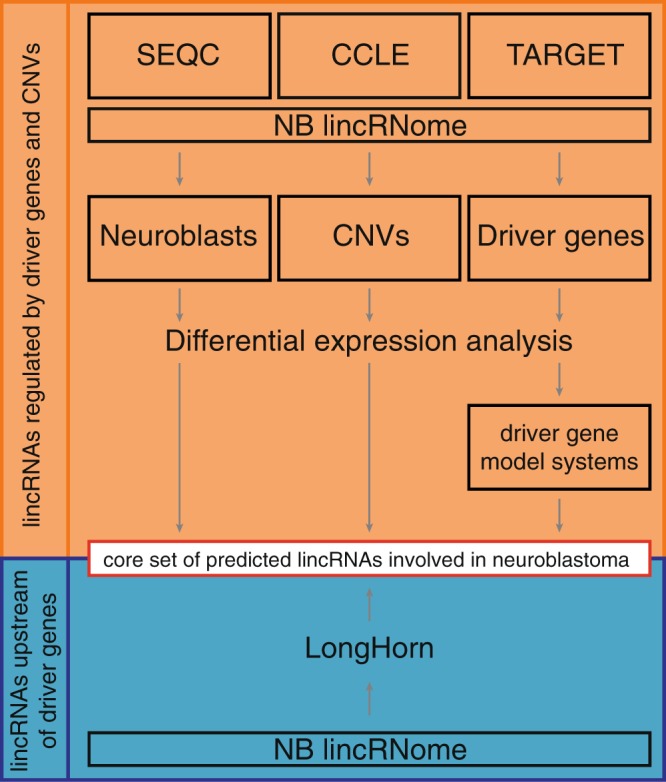
Included data sets and analyses in the study. The SEQC, CCLE and TARGET data sets were used to determine the NB lincRNome. The abundance of the robustly expressed lincRNAs in the lincRNome was used to compare expression between neuroblast and NB samples, primary tumor samples containing CNVs and samples without gains or deletions and NB tumors with and without mutations, amplifications or associations with NB driver genes. The regulation of lincRNAs correlated with NB driver genes was assessed in cellular perturbation models. To determine the involvement of lincRNAs in modulating the effect or regulation of these driver genes, we made use of a state-of-the-art algorithm called LongHorn. Combined, these analyses allow us to arrive at a core set of predicted NB associated lincRNAs.

In order to identify lncRNAs involved in key NB signaling pathways, we integrated RNA seq data from 497 primary NB tumor samples^32,33^, human fetal neuroblasts and various model systems with perturbation of driver gene activity. We present a comprehensive view on the NB lincRNome and prioritize lncRNAs up- and downstream of well-established NB driver genes, such as *MYCN*, *PHOX2B* and *ALK* (Fig. 1).

## Results

### The neuroblastoma lincRNome

**Figure 2:**
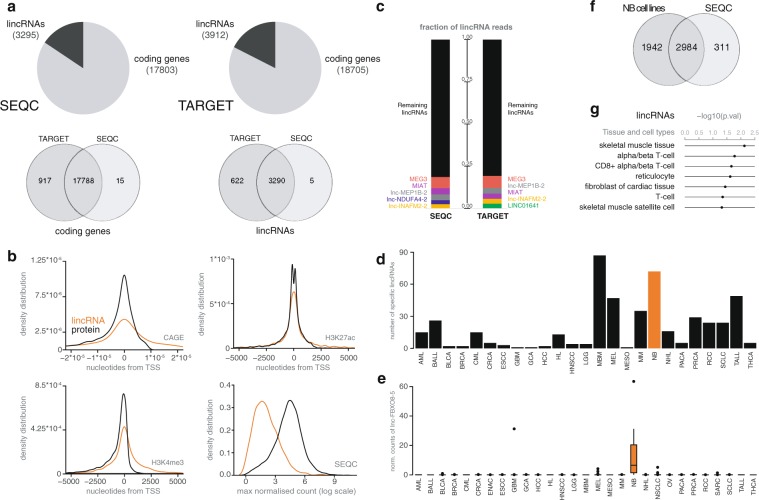
Establishing the NB lincRNome. (**a**) Based on Ensembl annotation, a set of 3295 lincRNAs was robustly expressed in the SEQC dataset, whereas 3912 lincRNAs were found in the TARGET dataset. A total of 3290 lincRNAs were expressed in both groups. (**b**) Density distribution plots showing distances of chromatin marks and CAGE peaks to the TSS and expression levels, for protein coding genes and lincRNAs. (**c**) Percentages of the lincRNA derived read counts for the top 5 expressed lincRNAs for the SEQC and TARGET datasets. (**d**) Bar plot showing the number of specific lincRNAs per cancer type. (**e**) Expression pattern of a randomly selected neuroblastoma specific lincRNA lnc-FBX08-5 across the different cancer types. (**f**) Overlap of expressed lincRNAs between NB cell lines and SEQC. (**g**) Fischer exact-test p-values (non-adjusted) for lincRNAs that are only expressed in the SEQC dataset and are associated with stromal cell types according to FANTOM5. (Cancer type abbreviations: AML: Acute monocytic leukemia; BALL: B-cell Acute Lymphoblastic Leukemia; BLCA: Bladder Carcinoma; BRCA: Breast Carcinoma; CML: Chronic Myelogenous Leukemia; CRCA: Colon Adenocarcinoma; ESCC: Esophageal Squamous Cell Carcinoma; GBM: Glioblastoma; GCA: Gastric Carcinoma; HCC: Hepatocellular Carcinoma; HL: Hodgkin Lymphoma; HSNCC: Head and Neck Squamous Cell Carcinoma; LGG: Brain Lower Grade Glioma; MBM: Medulloblastoma; MEL: Melanoma; MESO: Mesothelioma; MM: Plasma cell myeloma; NB: Neuroblastoma; NHL: Non-Hodgkin Lymphoma; PACA: Pancreatic Adenocarcinoma; PRCA: Prostate carcinoma; RCC: Renal Cell Carcinoma; SCLC: Small Cell Lung Carcinoma; TALL: T-cell Acute Lymphoblastic Leukemia; THCA: Thyroid gland carcinoma.)

To establish the NB lincRNome, we reanalyzed RNA seq data from 497 primary tumors, established by Zhang and colleagues as part of the SEQC study (further referred to as the SEQC dataset)^32,33^. Because the RNA sequencing data was unstranded (i.e. does not contain strand orientation information), we focused our analysis on lncRNAs classified as intergenic (lincRNAs) in Ensembl. This highly curated catalogue contains 7821 lincRNAs, of which 3295 were robustly expressed in NB tumors and hence define the NB lincRNome (Fig. 2A). The NB lincRNome was further validated in an independent RNA seq dataset of 148 primary tumors, generated by the TARGET consortium^34^, confirming expression for 3290 of the 3295 lincRNAs (Fig. 2A). To asses independent transcription of the lncRNAs, we’ve integrated publicly available CAGE seq (Cap Analysis Gene Expression) data from NB cell lines. Through CAGE seq, the 5′ end of a capped RNA molecule is sequenced, revealing the transcription start site of the transcript. By integrating this CAGE seq data using the Zipper plot^35^, we uncovered an enrichment of CAGE peaks at lincRNA transcription start sites (TSS), with 255 lincRNAs having a CAGE peak within +/−5 kb of their TSS (Fig. 2B). CAGE TSS enrichment is less pronounced compared to protein coding mRNAs, likely due to the fact that lincRNAs are less abundant than mRNAs (Fig. 2B) and public CAGE seq data is filtered based on a minimal expression cutoff^36^. Distribution of histone marks is more similar between lincRNAs and mRNAs, with 2933 and 2918 lincRNAs displaying a H3K4me3 or H3K27ac mark within +/−5 kb of their TSS, respectively. Although the majority of lincRNAs is weakly expressed, several are highly abundant. Up to 20% of all lincRNA reads are consumed by only 5 highly abundant lincRNAs in each of the investigated datasets (Fig. 2C). In contrast to adult cancers, where lincRNAs like *MALAT1*, *NEAT1* and *XIST* were most abundant^18^, we identified *MEG3* and *MIAT* as the most abundant lincRNAs in NB. While *MIAT* (also known as *GOMAFU*) has a dominant neuronal expression pattern^37,38^, it promotes growth and proliferation of multiple cancer types^39–41^. Interestingly, several other uncharacterized lincRNAs were found among the most abundant in NB tumors, including *lnc-MEP1B-2* and *lnc-INAFM2-2*. Moreover, we found *MEG3* and *MIAT* to be quite specific for NB cells (Supplemental Fig. 1). In fact, lincRNA expression patterns are known to exhibit remarkable cell-type specificity^1,6,7^. By comparing lincRNA expression in 917 cell lines representing 29 tumor types, we found NB among the tumor types with the highest number of specifically expressed lincRNAs (Fig. 2D,E). As several tumor-type specific lincRNAs have been shown to play important roles in tumor biology^42–45^, this information may thus help prioritize lincRNAs relevant for NB.

As tumor tissue biopsies to a variable degree are composed of stromal cells, we integrated the NB lincRNome with RNA seq data from NB cell lines to evaluate which lincRNAs are more likely to be derived from tumor or stromal cells. We found 2984 of 3295 lincRNAs from the NB lincRNome expressed in NB cell lines and 311 that were only detected in tumor biopsies (Fig. 2F). To evaluate whether the latter fraction is indeed more likely to be stromal cell derived, we used lincRNA tissue specificity data from the FANTOM consortium to look for cell or tissue ontology enrichments^3^. Various stromal cell types, including CD8+ alpha/beta T-cells were enriched among lincRNAs with expression restricted to tumor biopsies (Fig. 2G). These data suggest that a fraction of lincRNAs expressed in tumor tissue biopsies are indeed likely derived from stromal cells in these biopsies.

### lincRNAs are differentially expressed between NB tumors and neuroblasts

**Figure 3:**
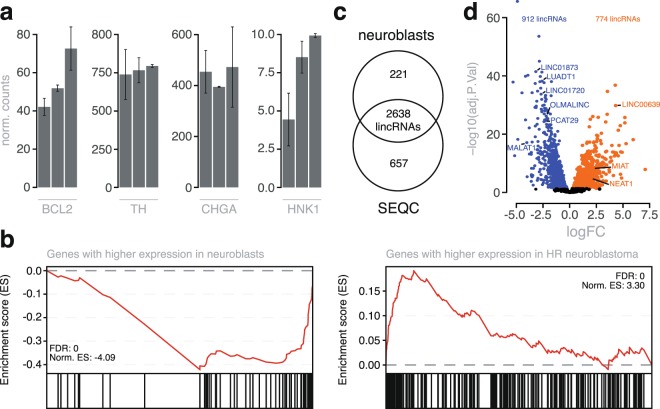
Establishing the neuroblast lincRNome. (**a**) Expression profiles of neural and chromaffin markers in the neuroblast samples (mean epxr. +/− SE). (**b**) GSEA results on a logFC ordered mRNA list, derived from differential expression analysis between neuroblasts and high-risk NB tumors, using neuroblast/HR NB specific gene sets. (**c**) Number and overlap of expressed lincRNAs in neuroblast and NB samples. (**d**) Volcano plot of differentially expressed lincRNAs between neuroblasts and high-risk neuroblastoma samples at q < 0.05. The orange dots represent upregulated genes (774 lincRNAs) in HR NB samples, whereas the blue dots depict genes with a lower abundance (912 lincRNAs).

NB predominantly exhibit a noradrenergic gene regulatory network including high expression levels of sympatho-adrenal lineage specific bHLH transcription factors such as PHOX2B and genes involved in dopamine synthesis such as tyrosine hydroxylase. We previously isolated and determined the transcriptome using gene expression arrays on microdissected human fetal neuroblasts and provided evidence for the presumed cell-of-origin for NB for these cells^46^. More recently, we have also generated human fetal neuroblast transcriptomes by RNAseq allowing to also explore expression of lincRNAs in normal reference cells versus NB cells. Detailed validation of the samples will be described elsewhere but importantly, well-established neuronal and chromaffin markers including *TH*, *CHGA*, *BCL2* and *HNK1* were expressed in all samples (Fig. 3A). To further validate the RNA seq data, we analyzed mRNA gene sets that were previously reported to be differentially expressed between neuroblasts and high-risk NB tumors^46^. Gene set enrichment analysis demonstrated a strong and significant enrichment of these signatures among up- and downregulated mRNAs between neuroblasts and high-risk NB tumors, supporting the validity of the expression dataset (Fig. 3B). We identified 2859 lincRNAs expressed in the neuroblast samples (Fig. 3C). The neuroblast lincRNome largely overlapped the NB lincRNome, with 2638 lincRNAs in common. Differential expression analysis revealed 774 and 912 lincRNAs that were significantly up- and downregulated in high-risk NB tumors compared to neuroblasts (Fig. 3D and Supplemental Table 1). Of interest, the highly abundant lincRNA *MIAT* showed a 4-fold upregulation in NB tumors compared to neuroblasts. Of the 774 lincRNAs upregulated in NB tumors, 447 were also expressed in NB cell lines and thus likely tumor derived.

### DNA copy number alterations drive lincRNA expression

Several studies have shown that DNA copy number alterations can drive lincRNA expression in cancer cells^47–49^. As high-risk NB tumors are characterized by recurrent segmental copy number alterations, we first evaluated lincRNA expression in regions with recurrent copy number gain (1q, 2p, 17q) and loss (1p, 3p, 11q). We found 23.7% of the neuroblastoma lincRNAome with significant positive correlation to their DNA copy number amplitudes (Supplemental Fig. 2). These results are in line with similar analysis in other cancer types and further support lincRNA dosage sensitivity.

**Figure 4:**
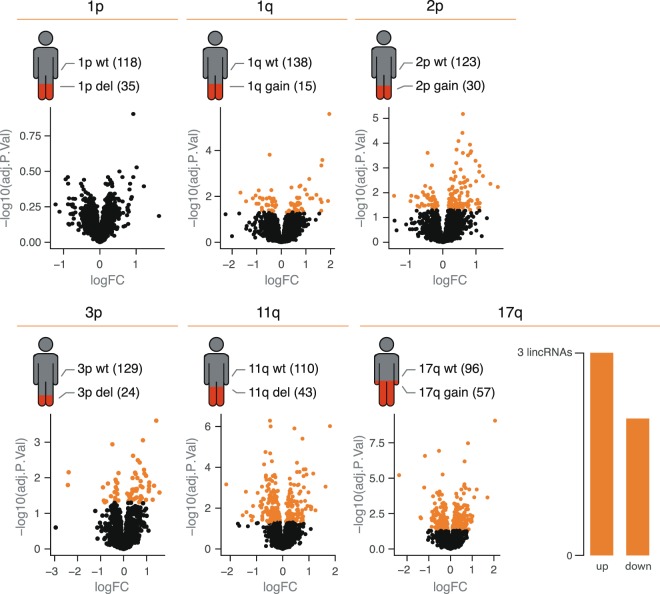
Altered expression levels of lincRNAs by copy number variations. Schematic representation of the number of cases with a copy number variation (1p deletion, 1q gain, 2p gain, 3p deletion, 11q deletion and 17q gain) present in our data set. The volcano plots show differentially expressed lincRNAs per copy number variation. Only 17q gain resulted in 5 robust significantly differentially expressed lincRNAs after iterative differential expression analysis (upregulated lincRNAs: *lnc-BRC1-2*, *LINC02432*, *lnc-RPS6KA4-3*; downregulated: *LINC02211*, *LINC01930*).

To evaluate whether segmental copy number alterations can indirectly impact lincRNA expression, we grouped tumors based on copy number status, followed by differential expression analysis (Fig. 4). In order not to confound the results with lincRNA expression differences driven by *MYCN* amplification, we excluded all *MYCN* amplified samples from the analysis. Differential lincRNAs were identified for each copy number alteration except for 1p deletions. However, when applying a more robust differential expression analysis, based on iterative subsampling, 17q gain was the only copy number alteration for which differential lincRNAs were identified. From the 5 lincRNAs differentially expressed between tumors with and without 17q gain, 3 were higher (*lnc-BRC1-2*, *LINC02432* and *lnc-RPS6KA4-3*) and 2 were lower (*LINC02211* and *LINC01930*) expressed in tumors with a 17q gain (Supplemental Table 2). These lincRNAs were also expressed in NB cell lines, confirming they are tumor derived. None of the upregulated lincRNAs were located on 17q, suggesting that 17q gain indirectly deregulates lincRNA expression in NB tumor cells.

### NB driver genes regulate lincRNA expression

NB tumors are characterized by low mutation rates^27,34,50^. As a consequence, the identification of oncogenic drivers has been challenging. *MYCN* amplification, activating mutations in the *ALK* receptor tyrosine kinase, *TERT* rearrangements, inactivating *ATRX* mutations and dominant negative mutations in *PHOX2B* are among the most recurrent genetic events that drive oncogenic signaling and tumor formation^23,25,27,51–54^. Our aim was to evaluate to what extent several of these well-established driver genes (*MYCN*, *ALK*) and neuroblastoma identity genes (*PHOX2B*) impact lincRNA expression.

#### Identification of lincRNAs regulated by NB driver genes in primary tumor samples

**Figure 5:**
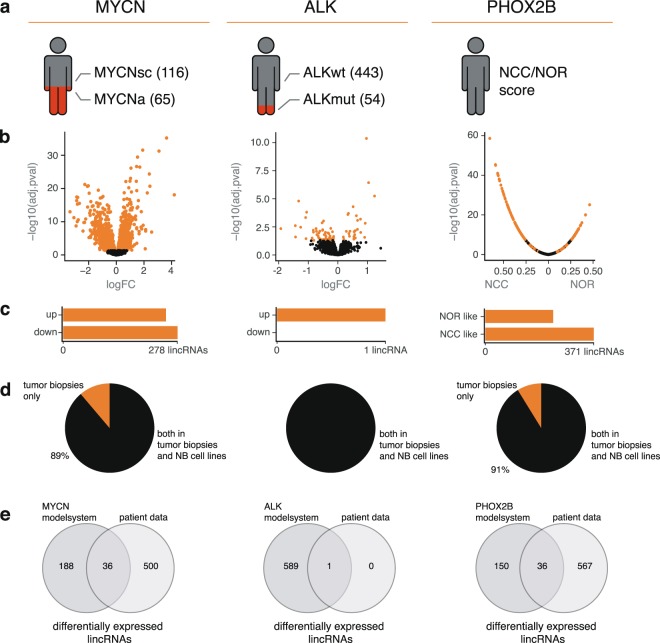
Regulation of lincRNAs by key driver genes. (**a**) Depiction of number of samples in our dataset for the three subtypes of genomic aberrations. (**b**) Volcano plot showing differentially expressed lincRNAs using all samples mentioned in (a) at q < 0.05 (orange dots). In the case of *PHOX2B*, correlation coefficient and adjusted p-value are represented. Here, the orange dots represent genes with opposing signs and significance for their correlation with the CRC scores. (**c**) Number of differentially expressed lincRNAs for *MYCN* and *ALK*. The far-right bar plot represents the number of lincRNAs found to be significantly correlated with both the *PHOX2B* CRC and *JUN*/*FOS* CRC in opposing directions. (**d**) Percentage of differentially expressed lincRNAs that are expressed in both the CCLE NB cell lines and the SEQC data set, or solely in the tumor biopsies. (**e**) Overlap of differentially expressed lincRNAs found in the SEQC analysis and after perturbation of the driver genes in the corresponding model systems (p < 0.05).

For *MYCN* and *ALK*, this was evaluated by grouping tumor samples based on driver gene status (i.e. amplified and mutated respectively) followed by differential lincRNA expression analysis (Fig. 5A,B). While *MYCN* amplification status was available for each tumor sample, *ALK* mutation status was not. *ALK* mutation status was therefore determined based on RNA seq data. We identified 54 tumors with missense mutations in the *ALK* gene, with the R1275Q and F1174L mutations as the most frequent ones (Supplemental Fig. 3). Differential mRNAs in tumors with varying *MYCN* or *ALK* status were strongly enriched for established mRNA gene sets previously shown to be regulated by *MYCN* or *ALK* (Supplemental Figs 3 and 4), thus validating our approach. Differential lincRNA expression analysis resulted in 1511 (773 upregulated and 738 downregulated) and 80 (55 upregulated and 25 downregulated) lincRNAs for *MYCN* and *ALK*, respectively (Fig. 5B and Supplemental Tables 3 and 4). When applying a more robust differential lincRNA expression analysis, 536 and 1 differential lincRNA(s) for *MYCN* and *ALK* respectively were retained for further analysis (Fig. 5C and Supplemental Tables 3 and 4). While the *ALK* associated lincRNA was also expressed in NB cell lines, expression of up to 11% of *MYCN* associated lincRNAs was restricted to NB tumor samples (Fig. 5D). The latter suggests these lincRNAs may be derived from stromal cells whose abundance or composition differs between tumors with and without *MYCN* amplification. To evaluate this hypothesis, we determined immune cell type fractions in each tumor through deconvolution of the mRNA expression data. We found a clear and significant difference in immune cell infiltration between *MYCN* amplified and *MYCN* single copy tumors for naïve B-cells, CD8+ T-cells and resting NK cells (Supplemental Fig. 5). These results confirm previous observations^55^ and support our hypothesis that a fraction of lincRNAs associated with *MYCN* amplification status are derived from infiltrating immune cells.

**Figure 6:**
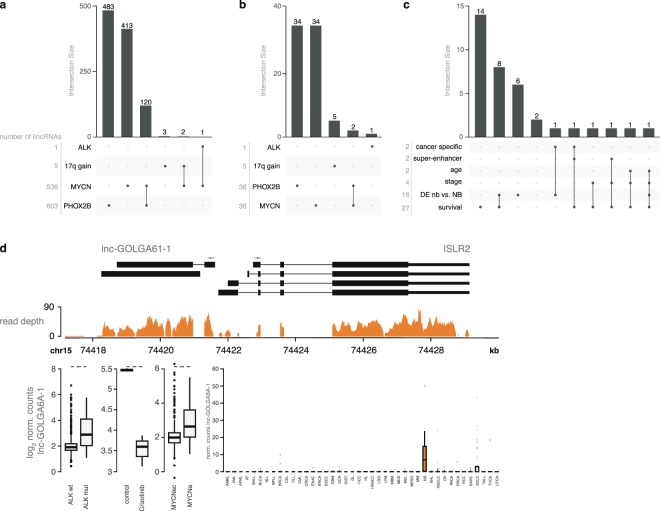
Association of lincRNAs with genetic and clinical parameters. (**a**) Number of lincRNAs regulated by one or more driver gene(s) or differentially expressed upon copy number variation in the patient samples. (**b**) Number of lincRNAs regulated by one or more driver genes or differentially expressed upon copy number variation, for lincRNAs differentially expressed in both the patient samples and model systems. (**c**) Number of lincRNAs regulated by MYCN, associated with clinical and genetic features. (**d**) Representation of the genomic locus and RNAseq data of *lnc-GOLGA6A-1*. *lnc-GOLGA6A-1* expression levels in *ALK* wild type and mutated samples, and *MYCN* amplified and single copy samples are shown, together with expression of the lincRNA upon crizotinib treatment, an ALK inhibitor. The boxplot shows the expression pattern of *lnc-GOLGA6A-1* across the different cancer types.

To identify *PHOX2B* associated lincRNAs, an alternative strategy based on two gene expression scores was applied. The first score reflects the activity of the *PHOX2B* core regulatory circuit (CRC) defining the noradrenergic cell state (NOR score). The second score reflects the activity of the *JUN*-*FOS* CRC, defining the neural crest cell state (NCC score) (Supplemental Fig. 6)^56^. As the activity of both CRC inversely correlates, *PHOX2B* associated lincRNAs were defined as lincRNAs that positively and negatively correlate to the NCC and NOR score, respectively, or vice versa. A total of 603 lincRNAs for which such relationship was identified were prioritized for further analysis (Fig. 5B,C and Supplemental Table 5). Similar to *MYCN*, 9% of these lincRNAs are not expressed in NB cell lines (Fig. 5D). We observed significant correlations between tumor NCC/NOR scores and the percentage of immune cells in the tumor biopsies, again suggesting that a fraction of these lincRNAs are stromal derived (Supplemental Fig. 7). Interestingly, 120 of the NCC/NOR associated lincRNAs were also differentially expressed in *MYCN* amplified tumors (Fig. 6A). Further, 2 and 1 lincRNA(s) that were differentially expressed in *MYCN* amplified tumors were also differentially expressed in tumors with a 17q gain or an *ALK* mutation, respectively.

#### Integration of driver gene model systems to validate lincRNA regulation

To evaluate which of the selected lincRNAs are regulated (directly or indirectly) by these driver genes, we used both in house generated data as well as publicly available RNA seq data for *MYCN*, *ALK* and *PHOX2B* perturbation model systems. For *MYCN* and *PHOX2B*, we applied inducible model systems containing a shRNA construct against *MYCN* (IMR5-75-shMYCN-TR)^57^ or *PHOX2B* (CLB-GA-shPHOX2B)^56^ or a *MYCN* overexpression construct^58^. For *ALK*, the *ALK* mutant NB cell line CLBGA was treated with the ALK inhibitor crizotinib. Out of 536 lincRNAs differentially expressed in *MYCN* amplified vs *MYCN* single copy tumor samples, 36 were also perturbed in at least one of two *MYCN* model systems (Supplemental Table 6). For *ALK* and *PHOX2B*, 1/1 and 36/603 differential lincRNAs were also regulated in the respective model systems (Fig. 5E and Supplemental Tables 7 and 8). These results demonstrate that NB driver genes can (directly or indirectly) regulate lincRNA expression. The majority of these lincRNAs appear to be regulated by a single driver gene (Fig. 6B), with only a small subset affected by multiple drivers (i.e. *PHOX2B* and *MYCN*). We found driver gene regulated lincRNAs to be strongly associated with NB patient survival and often differentially expressed between NB tumors and neuroblasts (Fig. 6C and Supplemental Fig. 8). For instance, 27/36 *MYCN* regulated lincRNAs are significantly associated with patient survival and 16 are differentially expressed between neuroblasts and high-risk NB tumors (Supplemental Table 9). Occasionally, these lincRNAs are located within a super-enhancer or display a NB-specific expression profile. In total, 14 lincRNAs are associated with multiple parameters, increasing their potential importance in NB biology.

One example is *lnc-GOLGA61-1*, a divergent lincRNA upstream of the *ISLR2* gene. *lnc-GOLGA61-1* is upregulated in tumor samples harboring an *ALK* mutation or a *MYCN* amplification. Treatment with crizotinib strongly represses *lnc-GOLGA61-1* expression, suggesting *ALK* is involved in *lnc-GOLGA61-1* regulation. Of interest, *lnc-GOLGA61-1* and *ISLR2* expression are strongly correlated in NB tumors (Supplemental Fig. 9). *ISLR2* is an interaction partner of *NTRK1* and *RET*, both involved in regulating NB differentiation, and *RET* has been shown to be activated by mutant *ALK*^59,60^. Expression of *lnc-GOLGA61-1* and *ISLR2* is restricted to NB cells as evidenced by RNA seq data of the CCLE cohort^61^ (Fig. 6D and Supplemental Fig. 10).

### LincRNAs as upstream regulators of neuroblastoma driver genes

#### LincRNAs as modulators of driver gene activity

**Figure 7:**
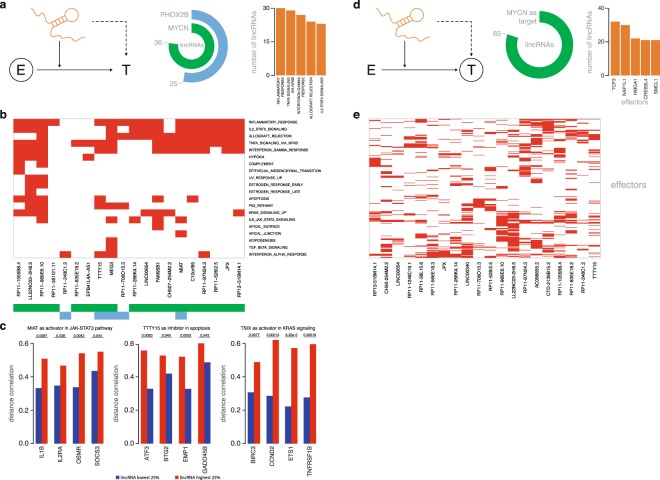
Identification of lincRNAs as modulators of activity or expression of driver genes. (**a**) Schematic representation of the investigated lincRNAs, modulating the activity of the effector proteins, MYCN and PHOX2B. The circular plots display the number of lincRNAs, expressed in NB cell lines, found to be modulators of MYCN and PHOX2B targets. Bar plots show the top 5 hallmarks that were significantly enriched (Fisher exact test, p < 0.001). (**b**) The heatmap visualizes the clustering of the significantly enriched hallmarks for the top 20 modulating lincRNAs. (**c**) Differences in distance correlation between the samples of low and high abundance of the lincRNAs. The presented targets are genes enriched in the hallmark gene sets. (**d**) Schematic representation of the investigated lincRNAs, regulating the activity of the target protein MYCN. The circular plots display the number of activating and inhibiting lincRNAs modulating *MYCN* expression. The top 5 effectors targeting *MYCN* are shown in the bar plot. (**e**) Clustering of the top 20 regulating lincRNAs with MYCN as target is visualized in the heatmap.

The above described analyses demonstrate the impact of various NB driver genes on lincRNA expression. However, lincRNAs can potentially also function upstream of, or in concert with these driver genes. To uncover such lincRNAs, we applied LongHorn, a computational pipeline aimed at uncovering effector genes and target genes of individual lincRNAs^62^. The pipeline essentially considers lincRNAs as modulators of effector proteins (i.e. transcription factors (TF) or RNA-binding proteins (RBP)) or as indirect regulators of target gene expression (Fig. 7). To uncover these relationships, LongHorn integrates mRNA and lincRNA expression data with experimental data on RNA-RBP interactions (eCLIP), TF regulation (ChIP-seq and PWM) and lincRNA-DNA binding site prediction (triplex). As both *MYCN* and *PHOX2B* are transcription factors, we first evaluated if lincRNAs could modulate *MYCN* or *PHOX2B* activity. Only tumor derived (i.e. expressed in primary tumors and cell lines) lincRNAs with a median absolute deviation >0.1 in the SEQC dataset were considered. LongHorn uncovered 25 and 36 lincRNAs that were predicted to modulate *PHOX2B* and *MYCN* activity, respectively (Fig. 7A, Supplemental Table 9). Importantly, none of these lincRNAs correlate with *MYCN* or *PHOX2B* expression levels directly, excluding the possibility that such correlations may confound the results. LincRNAs predicted to modulate *MYCN* activity included - amongst others - *MIAT*, *TSIX* and *MEG3*. Of note, 7 lincRNAs were found to modulate both *MYCN* and *PHOX2B* activity. Targets that were predicted to be affected by lincRNA modulation with *MYCN* or *PHOX2B* as effectors were subsequently evaluated for enrichment of hallmark gene sets. We observed enrichments for, amongst others, the TP53 pathway (8 lincRNAs), STAT signaling (24 lincRNAs), KRAS signaling (14 lincRNAs) and the apoptosis pathway (9 lincRNAs) (Fig. 7B). We identified *TSIX* as a modulator of *MYCN* activity, driving expression of KRAS signaling genes including *BIRC3* and *CCND2* (Fig. 7C). Alternatively, *TTTY15* was found to modulate *MYCN* activity towards repression of the apoptosis pathway genes *GADD45B* and *BTG2* (Fig. 7C).

#### LincRNAs as regulators of driver gene activity

Next to direct modulation of *MYCN* or *PHOX2B* activity, lincRNAs may also modulate effectors of *MYCN*, *PHOX2B* or *ALK* expression (Fig. 7D). We identified 65 lincRNAs that were predicted to regulate *MYCN* expression through modulation of one or more effectors. Several effectors of *MYCN* expression, including *TCF3*, *NAP1L1*, *HMGA1* and *CREB3L4* were predicted to be modulated by multiple lincRNAs (Fig. 7D,E). No lincRNAs were predicted to regulate *PHOX2B* or *ALK* expression. Taken together, these analyses demonstrate that lincRNAs may indeed regulate or modulate the expression or activity of one or multiple NB driver genes.

## Discussion

We have evaluated RNA seq data of primary NB tumors, human fetal neuroblasts and various cellular perturbation model systems to reveal alterations in lincRNA expression patterns invoked by driver mutations, amplifications or DNA copy number variations. Through various prioritization strategies, we provide a core set of lincRNAs with a potential role in NB tumor biology, up- or downstream of the key NB driver genes *MYCN*, *ALK* and *PHOX2B*.

Integration of RNA seq data from primary tumors and cell lines revealed that a fraction of the lincRNAs expressed in tumor biopsies were not detected in cell lines. Cell lines are known to have higher levels of CpG hypermethylation than primary tumors^63,64^, potentially explaining why expression of certain lincRNAs was restricted to tumor samples. In addition, the tumor sample cohort is more heterogeneous in nature compared to the NB cell lines which are typically derived from high-risk tumors only. However, tumor biopsies also have a stromal component that is absent in cell lines, leading us to hypothesize that some of these lincRNAs are stromal in origin. Tissue ontology enrichment analysis provided support for this hypothesis. Moreover, we could demonstrate that stromal composition, and more specifically immune cell infiltration, correlated with *MYCN* amplification status and *PHOX2B* core regulatory circuit activity. This confounded our differential lincRNA expression analysis between *MYCN* amplified and *MYCN* single copy tumors and lincRNA *PHOX2B* CRC correlation analysis. As a result, many lincRNAs that were prioritized as differentially expressed or correlated, were undetected in NB cell lines and thus likely stromal cell derived. Cell type composition of tumor biopsies can be elucidated using computational deconvolution methods^65–67^. In addition, RNA seq data from various human cell types is becoming increasingly available^3^. Integrating this type of information when performing (differential) lincRNA (or mRNA) expression analysis on tumor biopsies could help elucidate the cell of origin of RNA molecules and assist in prioritizing key players in tumor biology.

By combining RNA seq data from primary tumors with model systems for *MYCN*, *ALK* and *PHOX2B*, we could demonstrate that each of these driver genes regulate a core set of lincRNAs. Whether these lincRNAs are regulated directly (e.g. through binding of *MYCN* or *PHOX2B* transcription factors in the lincRNA promoter) or indirectly remains to be determined. Several of these lincRNAs were found to be differentially expressed between NB tumors and precursor neuroblasts and/or associated with patient survival and disease stage. To further prioritize driver gene regulated lincRNAs, we evaluated their link with super-enhancers and NB expression specificity. Several lincRNAs that play a role in tumor biology, including *CCAT1-L* and *SAMMSON*^42,68^ are associated with these features. This core set of NB driver gene regulated lincRNAs should be further explored by genetic perturbation experiments to investigate their impact on the cellular and molecular phenotype.

As driver genes themselves could be under the control of one or more lincRNAs, we applied a state-of-the-art computational workflow aimed at prioritizing lincRNAs that modulate driver gene activity or expression. This resulted in 36 and 25 lincRNAs that modulate *MYCN* and *PHOX2B* activity, respectively. Functional associations between *MYC* and several lincRNAs predicted to modulate *MYCN* activity, have been demonstrated previously for *MEG3*^69^ and *TSIX*^70^ amongst others. *MIAT*, one of the most abundant lincRNAs in NB, was identified as a modulator of both *MYCN* and *PHOX2B*. Target genes that were affected as a result of this modulation were significantly enriched in the IL6-JAK-STAT3 pathway. Interestingly, *MIAT* has been described to enhance *STAT3* expression by acting as a molecular sponge for *miR-181b*^71^, a miRNA upregulated in *MYCN* amplified NB tumors^72^. *MIAT* was also identified, together with 48 additional lincRNAs, as a candidate to modulate the activity of effectors of *MYCN* expression. *TCF3*, *HMGA1* and *CREB3L4* were among the most recurrent effectors of *MYCN*. *HMGA1* is able to regulate *MYCN* expression in NB cells^73^, while *TCF3* has been shown to regulate *MYC* expression in colorectal cancer cells^74^. Moreover, *TCF3* has previously been identified as a master regulator in *MYCN* amplified NB tumors^75^.

In summary, we identified a comprehensive catalogue of lincRNAs up- and downstream of key NB driver genes. These lincRNAs could play an important role in tumor initiation and progression and may serve as a solid starting point for further experimental validation.

## Material and Methods

### Annotation and quantification

The TARGET fastq files were downloaded from the Genomics Data Commons Data portal. Kallisto (v0.42.4) was used to quantify gene expression in the samples, using the hg38 human assembly (GRCh38.p10), encompassing 37,297 lincRNA and 180,869 protein coding transcripts. Only long intergenic non-coding RNAs were considered, as RNA seq data for both data sets was unstranded. The transcripts were classified according to the Ensembl biotype annotation (GRCh38.p10).

### Cancer cell line encyclopedia

RNA sequencing data of the Cancer Cell Line Encyclopedia was reprocessed using Kallisto with the hg38 human genome assembly (GRCh38.p10). using this RNA seq data set to filter out lincRNAs not expressed in NB cell lines (i.e. tumor specific). Uberon and cell types assigned to lincRNAs were downloaded from FANTOM5^3^. Only lincRNAs with Ensembl gene IDs were selected. Enrichment of stromal cell types was determined based on tumor specific lincRNAs (Fisher Exact test, p < 0.05).

Specificity of genes was calculated based on a minimum fold change >3 between median expression of each cancer type per gene.

### Neuroblasts

Ethical approval was obtained for the collection of fetal adrenal glands from fetuses aborted for clinical reasons and informed consent was obtained for the use of all samples (Ethics committee Erasme Hospital, Brussels, Belgium; approval no.: OM021). All methods were carried out in accordance with relevant guidelines and regulations. Neuroblasts were isolated from 3 fetal adrenal glands from 13–16 week gestation embryos using laser capture microdissection. We extracted RNA from 6 neuroblast clusters and 3 areas of adjacent normal adrenal cortex as controls using the PicoPure kit (ARCTURUS). Samples were PCR amplified (SMART-Seq v4 ultra low input RNA kit, Takara Bio) and sequenced on the Illumina Hiseq 4000 platform to create a unique resource of neuroblast mRNA and lincRNA expression data.

### Copy number analysis

Copy numbers status was determined using array CGH. Copy number amplitudes (CNA) higher than 2.5 and lower than 1.5 were annotated as aberrant. Each segment was annotated by its corresponding chromosome arm, allowing classification according to known chromosome arm gains and deletions in NB tumors. To assess of dosage sensitivity of lincRNAs, a CNA was assigned to each gene per sample, based on its chromosomal location. Correlation with expression was calculated using Pearson’s method, p values were adjusted using the Benjamini-Hochberg method^76^ (q < 0.05).

### RNA sequencing data based mutation analysis

The SEQC and TARGET data set were aligned to the human hg19 assembly, using TopHat (v2.10). Mutations were identified by means of the Genome Analysis ToolKit (v3.2–2) using the RNA-seq best practices workflow. Only mutations in protein coding genes deemed damaging or possibly damaging by means of Polyphen and Sift, were retained. Variants with a prevalence of more than 0.1% according to gnomAD, having a total read-depth below 5 or a read-depth for the mutant allele below 3, were filtered out.

### Differential expression analysis

Limma voom (v3.36.5) was used to assess differential expression between neuroblast and high-risk neuroblastoma samples, mutated and non-mutated samples for *ALK*, and amplified and non-amplified samples with INSS stage 4 for *MYCN* and gain/deletions and wild type samples for the CNVs, for genes expressed in at least half of the SEQC samples. Genes were classified as differentially expressed based on their adjusted p-value (q < 0.05). For a more robust differential expression analysis for *ALK* and *MYCN* differentially expressed genes, the SEQC data set was divided into two subgroups, having an equal number of mutated or amplified samples. Differential expression analysis was performed for both subgroups and genes differentially expressed in both groups were identified (q < 0.05). This workflow was repeated 100 times, and only genes differentially expressed in more than 80 of the repeats were classified as being truly differentially expressed genes. Differential expression in the model systems was calculated using limma voom, with a threshold of p < 0.05 (non-adjusted p-value).

### Gene set enrichment analysis

Gene set enrichment analysis^77^ was performed using the Java GSEA application of the Molecular Signatures Database (*1000 permutations, classic analysis*). To validate differentially expressed genes between high-risk NB samples and neuroblasts, a list of mRNAs was analyzed through pre-ranked GSEA, using gene sets built out of known differentially expressed genes between these two groups of samples^46^. GSEA was also performed on mRNA lists resulting from differential expression analysis between *MYCN* amplified and *MYCN* single copy tumors (INSS stage 4) and mRNA list from treated and untreated *MYCN* model systems, using all curated gene sets in the Molecular Signatures Database (Supplemental Figure 11). For the GSEA on mRNA list from differential expression analysis for the CNVs, only the positional genesets were used (Supplemental Fig. 12). In the case of *ALK*, no gene sets are available. As such, we created our own gene sets based on a validated *ALK* mRNA signature^59^. Significant enrichment was defined at FDR < 0.05. All mRNA lists were ordered based on the log-transformed fold change.

### Defining stromal cell composition

CIBERSORT^67^, a computational method to estimate cell type fractions from bulk RNA-seq data, was used to define the cell type composition of the primary tumors. Gene expression data sets with raw counts were used as input. The algorithm (v1.04) was run in R (v3.5.0) with the default signature matrix at 100 permutations. Statistical significance between the *MYCN* amplified and *MYCN* single copy subset was calculated with a Mann-Whitney test. For samples associated with the *PHOX2B* or the *JUN*/*FOS* core regulatory circuit, correlations of the percentages of immune cell types and the ranksums were calculated with Spearman’s rank correlation method. P-values were adjusted using the Benjamini-Hochberg method^76^. Significance cut-off was set at q < 0.05.

### Cell line perturbation models

Four neuroblastoma model systems were used in this study. For *MYCN*, publicly available RNA seq data sets for IMR5-75-shMYCN-TR (ArrayExpress E-MTAB-6568) and SHEP-MYCN-TET (Gene Expression Omnibus GSE83327) cells were used. CLBGA-shPHOX2B cells^56^ were treated with doxycycline for 5 days (n = 2), together with a shControl cell line (n = 2) (Supplemental Fig. 13). *ALK* mutant CLBGA cells were treated with *ALK* inhibitor crizotinib at a concentration of 500 nM for 24 hours. Over the course of 3 weeks, matched treatment of 1 sample and 1 control sample was performed per week.

### cDNA library prep and sequencing

Total RNA was extracted from the CLB-GA cell line for the ALK model system using TRIzol Reagent (Invitrogen) and the miRNeasy Mini Kit (Qiagen). For the CLB-GA-shPHOX2B system, total RNA was extracted from fresh cells using TRIzol® Reagent (Invitrogen) and the AllPrep DNA/RNA Mini Kit (Qiagen). All samples were subjected to quality control on a Bioanalyzer instrument and all RNA exhibited a RIN (RNA Integrity Number) >8. All RNA sequencing libraries were prepared from 200 ng of total RNA using the Illumina TruSeq Stranded mRNA Library preparation kit. Kappa qPCR quantification was used to perform equimolar pooling. The concentration of the pooled library was measured with Qubit. Sequencing of 1.2 pM of pooled library was performed with the Illumina NextSeq 500 instrument using 2 × 75 cycles (paired-end) for all samples (high output sequencing kit). Transcripts were quantified by means of Kallisto using the human GRCh38 transcriptome as a reference.

### Survival analysis

Overall survival analysis was performed on the SEQC data set using a Kaplan-Meier analysis. The curves were created by dichotomizing the RNA-expression data, using the median expression value as a cutoff. The log-rank test was used to compare the two curves and generate a significance level of the impact of expression on overall survival for each gene. Multiple testing correction was performed using the Benjamini-Hochberg method. Genes with q < 0.05 were considered to be associated with survival.

### Super-enhancers

H3K27ac ChIP-sequencing and super-enhancer analysis using LILLY was performed as described^78^ (Supplemental Fig. 14). A gene was classified to be located in a super-enhancer if that region was called in a minimum of 14 different NB cell lines. The lincRNAs were ordered based on their mean rank over all NB cell lines.

### Longhorn

LongHorn searches for sequence patterns in proximal promoters that are predictive of RNA-DNA triplex structures identified by Triplexator^79^, and expression-based evidence for modulation of transcription factor (TF) activity. To predict modulation, we first collected candidate TF-target pair interactions and then tested for evidence of their modulation. For all candidate TF-target pairs, we required that each TF-target candidate has a significant nonlinear correlation (p < 1E-11) as estimated by distance correlation (dCor), and either TF binding evidence from ENCODE ChIP-Seq assays or predicted interactions based on published TF binding-site motifs. To collect evidence for the modulation of these TF-target interaction candidates, we used delta dCor within a triplet composed of a lncRNA, a TF, and a protein-coding target. Specifically, for each lncRNA, we partitioned all tumor samples into four groups based on the expression profile of this lncRNA, from lowest to highest. To avoid circularity, for each triplet, we added an independence constraint by requiring that the lncRNA was not correlated with the TF (p > 0.1) and a range constraint by requiring a minimum of 2x fold-change between the lncRNA’s average expression in the two sets (low vs high). Then, comparing the sample groups with highest and lowest lncRNA expression, we required a nonparametric p < 0.05 for the delta dCor between the TF and the target against a bootstrapping-based null hypothesis. These p-values were integrated across all significant triplets using Fisher’s method to identify significant lncRNA-target pairs at an adjusted p < 0.01. Enrichment of the cancer hallmarks gene sets of the Molecular Signatures Database was calculated using Fisher’s exact test (q < 0.05)^80^. The top 20 lincRNAs modulating *MYCN* or *PHOX2B* effect were selected based on the minimum calculated adjusted p-value, regardless of the specified effector. LincRNAs with *MYCN* as target in the heatmap were selected based on the minimum p-value. All selected lincRNAs were expressed in NB cell lines.

## Supplementary information

Supplemental Figures

Supplemental table 1

Supplemental table 2

Supplemental table 3

Supplemental table 4

Supplemental table 5

Supplemental table 6

Supplemental table 7

Supplemental table 8

Supplemental table 9

Supplemental table 10

## Data Availability

RNA sequencing data of the ALK and PHOX2B model systems have been deposited in GEO with the accession codes GSE124450 and GSE124451.
