# Author Correction: Integrative analysis identifies lincRNAs up- and downstream of neuroblastoma driver genes

**DOI:** 10.1038/s41598-019-46785-6

**Published:** 2019-07-17

**Authors:** Dries Rombaut, Hua-Sheng Chiu, Bieke Decaesteker, Celine Everaert, Nurten Yigit, Agathe Peltier, Isabelle Janoueix-Lerosey, Christoph Bartenhagen, Matthias Fischer, Stephen Roberts, Nicky D’Haene, Katleen De Preter, Frank Speleman, Geertrui Denecker, Pavel Sumazin, Jo Vandesompele, Steve Lefever, Pieter Mestdagh

**Affiliations:** 1 Center for Medical Genetics, Ghent University, Ghent, 9000 Belgium; 2 Cancer Research Institute Ghent (CRIG), Ghent, 9000 Belgium; 3 Texas Children’s Cancer Center, Baylor College of Medicine, Houston, TX 77030 USA; 4 Institut Curie, PSL Research University, Inserm U830, Equipe Labellisée contre le Cancer, F-75005 Paris, France; 5 SIREDO: Care, Innovation and Research for Children, Adolescents and Young Adults with Cancer, Institut Curie, F-75005 Paris, France; 6 Center for Molecular Medicine Cologne (CMMC), University of Cologne, 50931 Cologne, Germany; 7 Department of Experimental Pediatric Oncology, University Children’s Hospital of Cologne, Medical Faculty, University of Cologne, 50937 Cologne, Germany; 8 Department of Pediatrics, Memorial Sloan Kettering Cancer Center, New York, NY USA; 9 Hôpital Erasme, Cliniques Universitaires de Bruxelles, Bruxelles, 1070 Belgium

Correction to: *Scientific Reports* 10.1038/s41598-019-42107-y, published online 05 April 2019

The original version of this Article contained a typographical error in the spelling of the author Isabelle Janoueix-Lerosey, which was incorrectly given as Isabelle Janoueix. This has now been corrected in the PDF and HTML versions of the Article, and in the accompanying Supplementary Information files.

