# Supplementary material for: Integrative analysis identifies lincRNAs up- and downstream of neuroblastoma driver genes
Source: Sci Rep. 2019 Apr 5;9:5685. doi: 10.1038/s41598-019-42107-y (PMC6451017; doi:10.1038/s41598-019-42107-y)
Supplement: Supplementary file 1 — Supplemental Figures [file 41598_2019_42107_MOESM1_ESM.pdf]

Integrative analysis identifies lincRNAs up- and downstream of neuroblastoma driver genes

Dries Rombaut[1,2], Hua-Sheng Chiu[3], Bieke Decaesteker[1,2], Celine Everaert[1,2], Nurten Yigit[1,2], Agathe Peltier[4,5], Isabelle Janoueix-Lerosey[4,5], Christoph Bartenhagen[7], Matthias Fischer[6,7], Stephen Roberts[8], Nicky D'Haene[9], Katleen De Preter[1,2], Frank Speleman[1,2], Geertrui Denecker[1,2], Pavel Sumazin[3], Jo Vandesompele[1,2], Steve Lefever[1,2], Pieter Mestdagh[*1,2]

1. Center for Medical Genetics, Ghent University, Ghent, 9000, Belgium

2. Cancer Research Institute Ghent (CRIG), Ghent, 9000, Belgium

3. Texas Children's Cancer Center, Baylor College of Medicine, Houston, TX 77030, USA

4. Institut Curie, PSL Research University, Inserm U830, Equipe Labellisée contre le Cancer, F-75005, Paris, France.

5. SIREDO: Care, Innovation and Research for Children, Adolescents and Young Adults with Cancer, Institut Curie, F-75005, Paris, France.

6. Center for Molecular Medicine Cologne (CMMC), University of Cologne, 50931 Cologne, Germany

7. Department of Experimental Pediatric Oncology, University Children's Hospital of Cologne, Medical Faculty, University of Cologne, 50937 Cologne, Germany

8. Department of Pediatrics, Memorial Sloan Kettering Cancer Center, New York, NY, United States of America 9. Hôpital Erasme, Cliniques Universitaires de Bruxelles, Bruxelles, 1070, Belgium

* Corresponding author: Pieter.Mestdagh@UGent.be

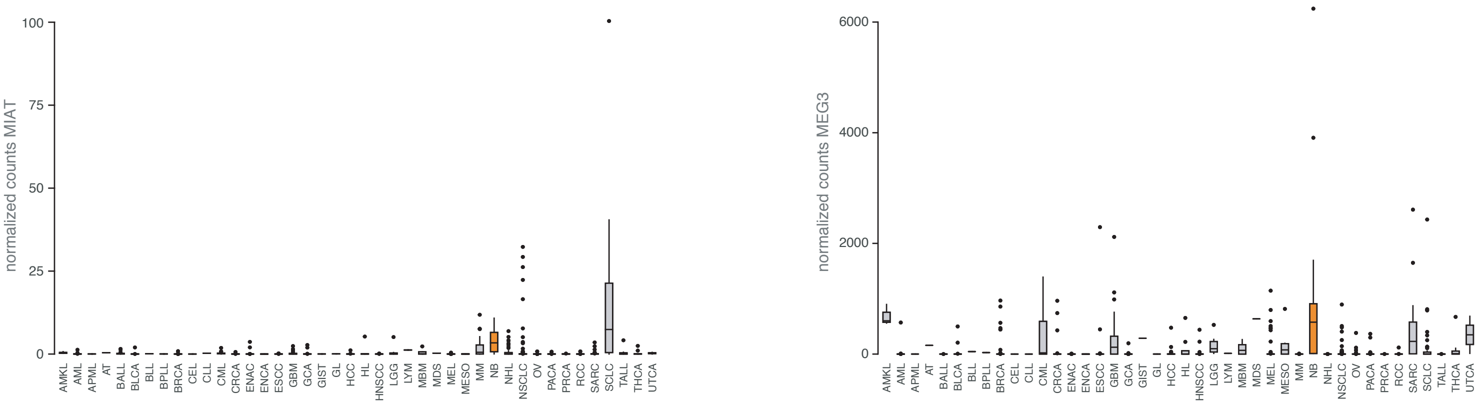

Supplemental Figure 1: Boxplot showing expression levels of *MIAT* and *MEG3* across different cancer cell lines.

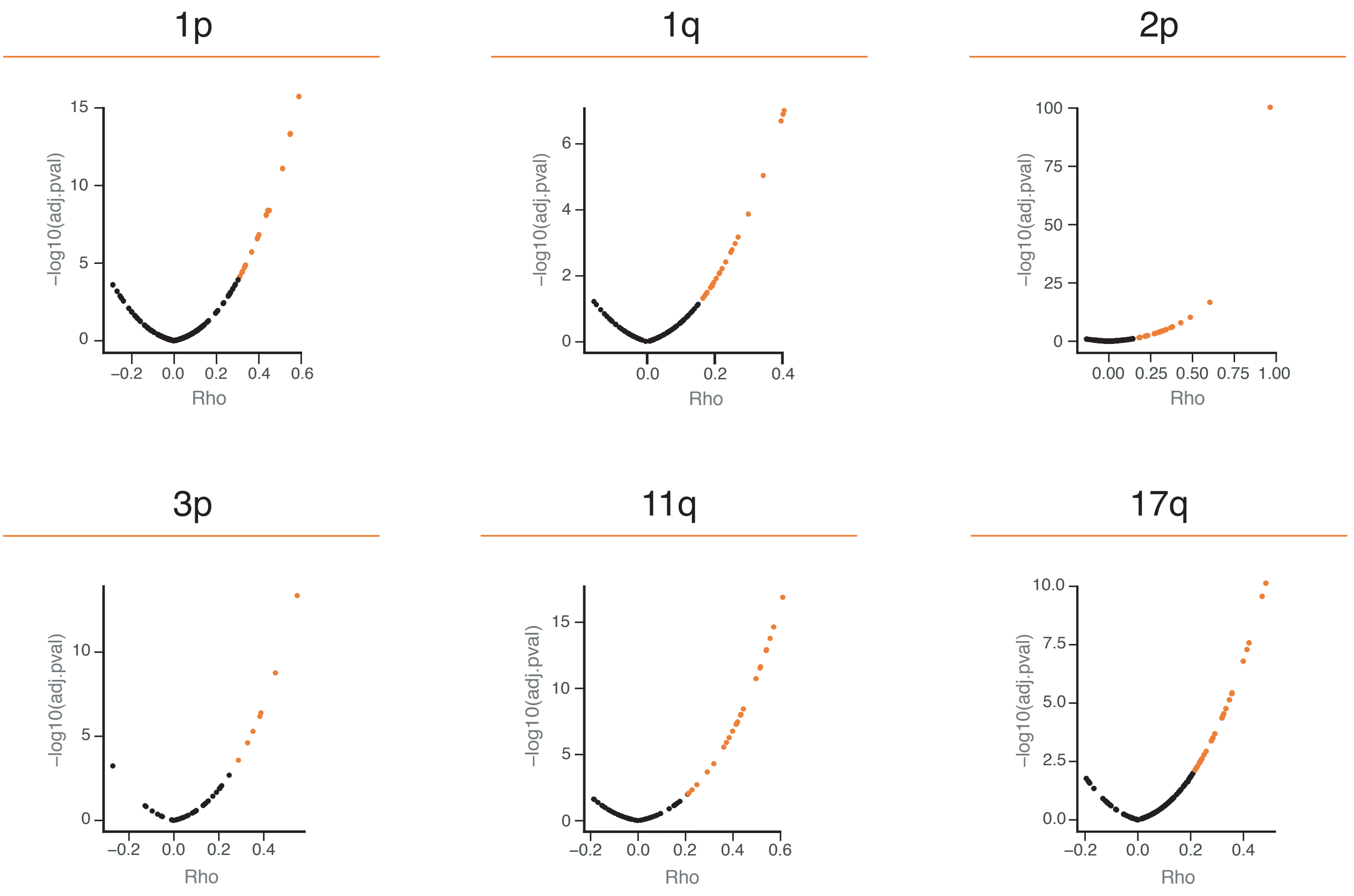

Supplemental Figure 2: Volcano plots with correlation coefficients and p-values for lincRNA expression and copy number variations, for lincRNAs situated on the respective chromosomal arm.

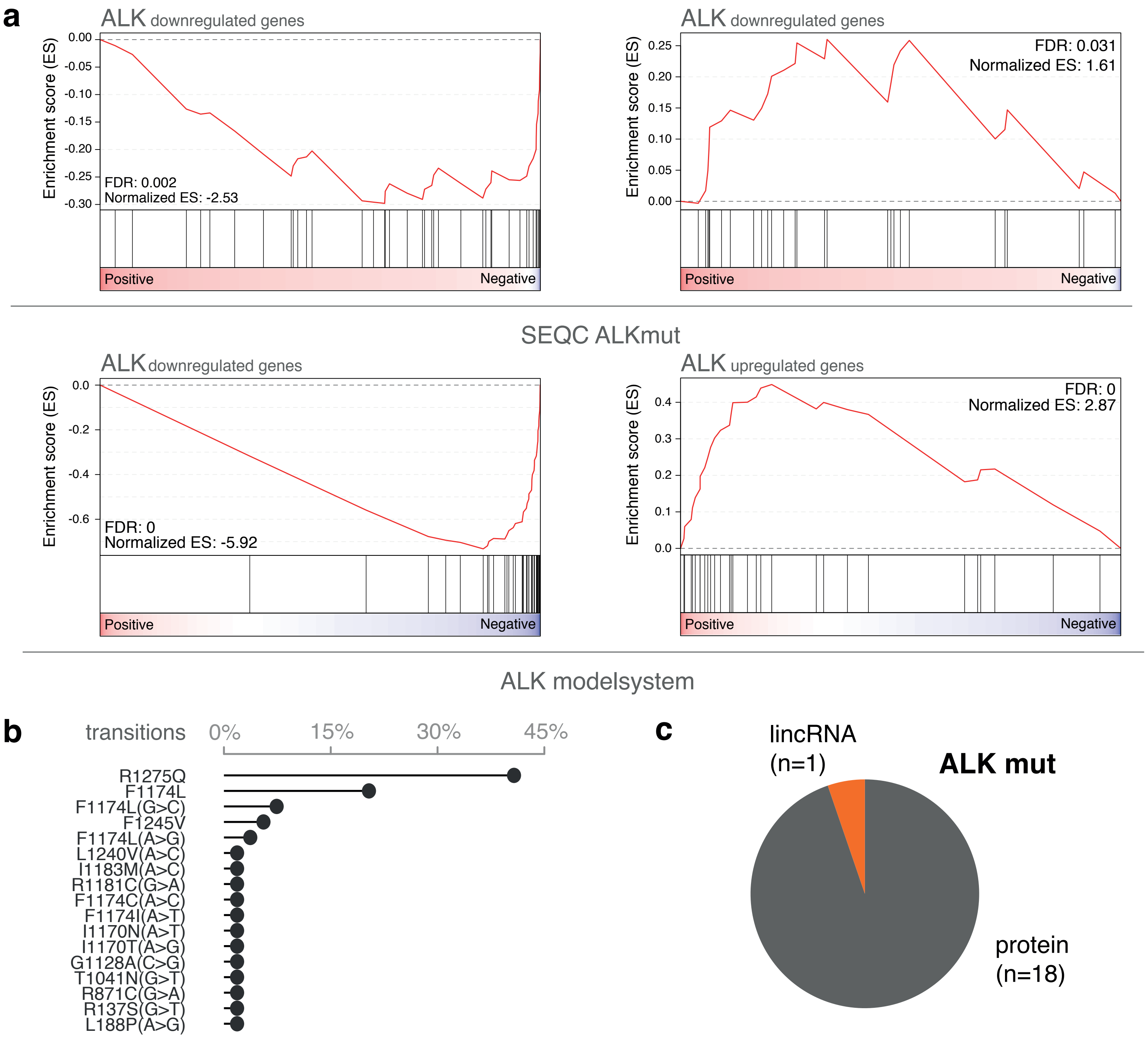

Supplemental Figure 3: a) GSEA plots for the differentially expressed genes between *ALK* mutated and wild type samples and *ALK* treated and control samples, respectively. b) Percentage of samples harboring selected mutations found in the SEQC data. c) Number of differentially expressed lincRNAs and mRNAs after robust differential expression analysis.

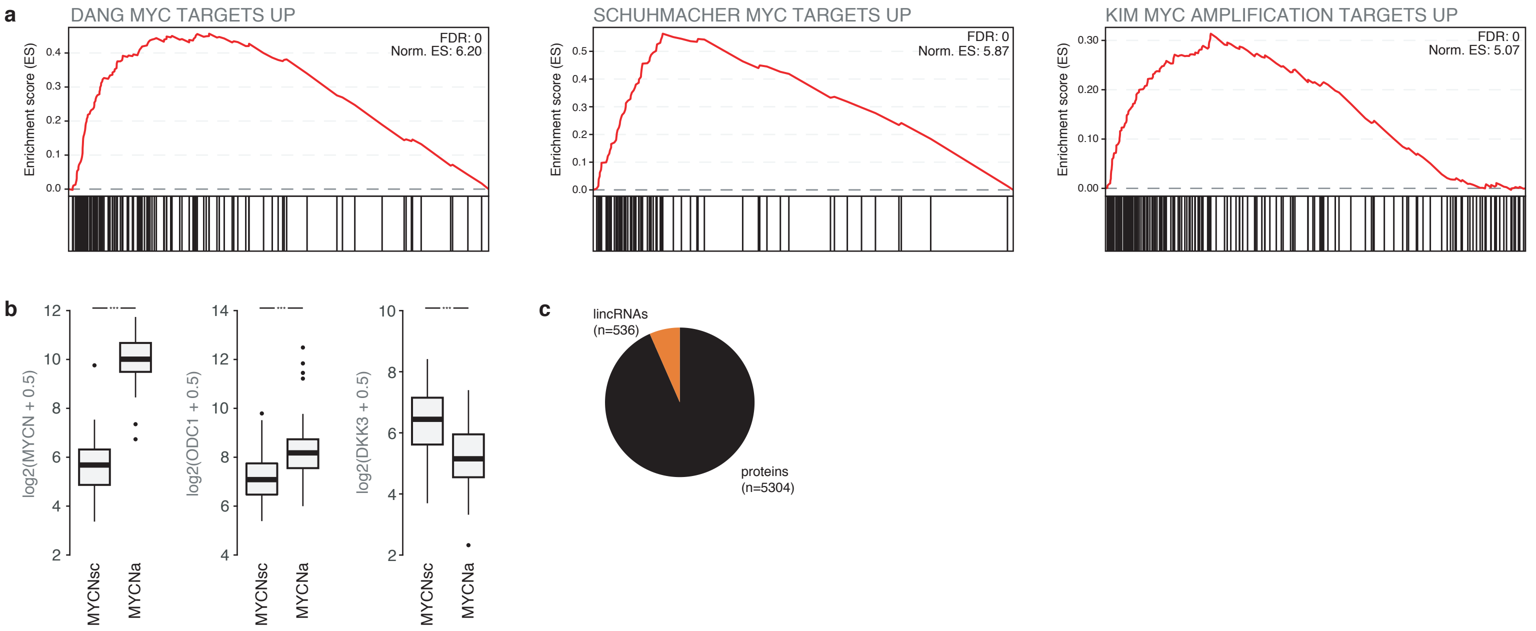

Supplemental Figure 4: a) GSEA showing enrichment for MYC(N) targets, after differential expression analysis between *MYCN* amplified and *MYCN* single copy samples. b) Boxplot displaying the expression levels of *MYCN* and two bona fide target genes, *ODC1* and *DKK3*. c) Number of lincRNAs and mRNAs robustly differentially expressed.

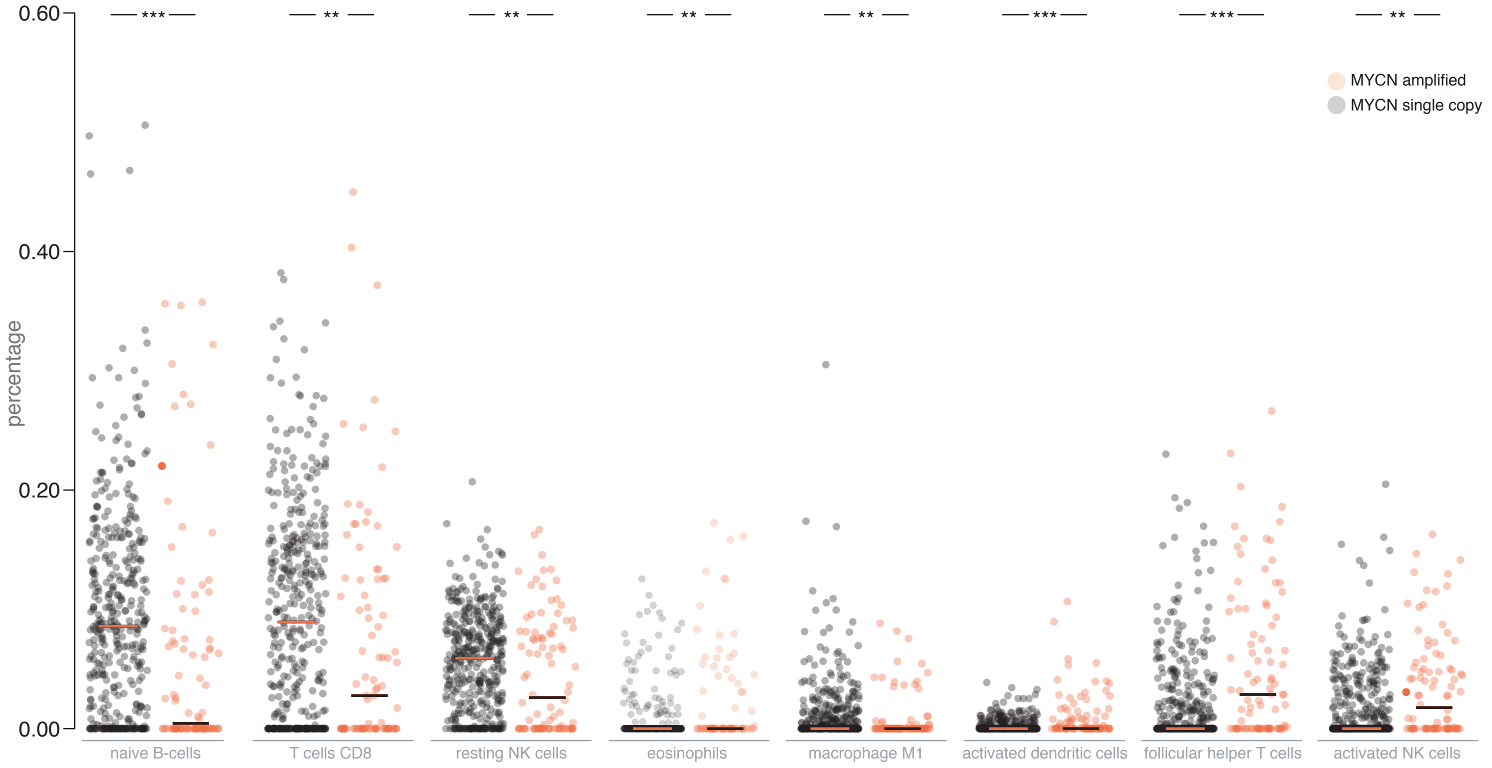

Supplemental Figure 5: The percentage of immune infiltration of 8 immune cell types between *MYCN* amplified and *MYCN* single copy tumors. Statistical significance was calculated using a Mann-Whitney test (q<0.05).

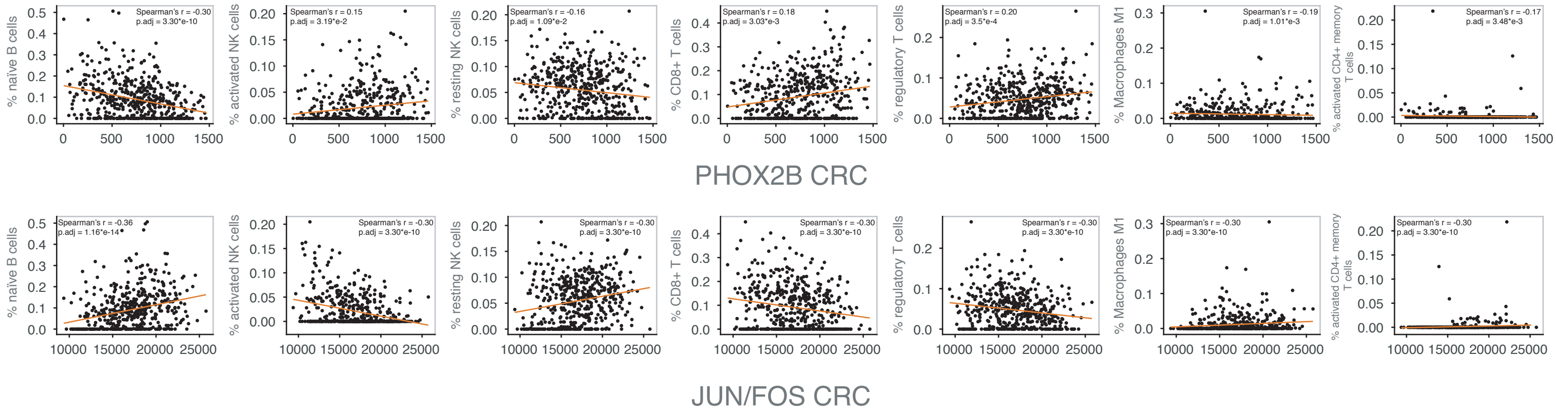

Supplemental Figure 6: Correlation plots of 7 immune cell types with significant correlation with the *PHOX2B* CRC or *JUN/FOS* CRC score. Correlation coefficient and p-value were determined using Spearman's method.

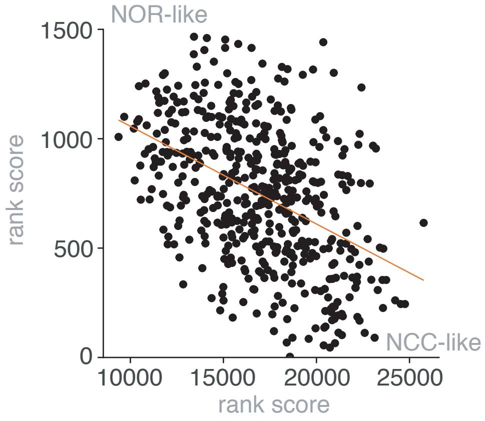

Supplemental Figure 7: Scatterplot visualizing the inverse correlation between the rank score calculated for the *PHOX2B* (NOR-like) and *JUN/FOS* CRC (NCC-like).

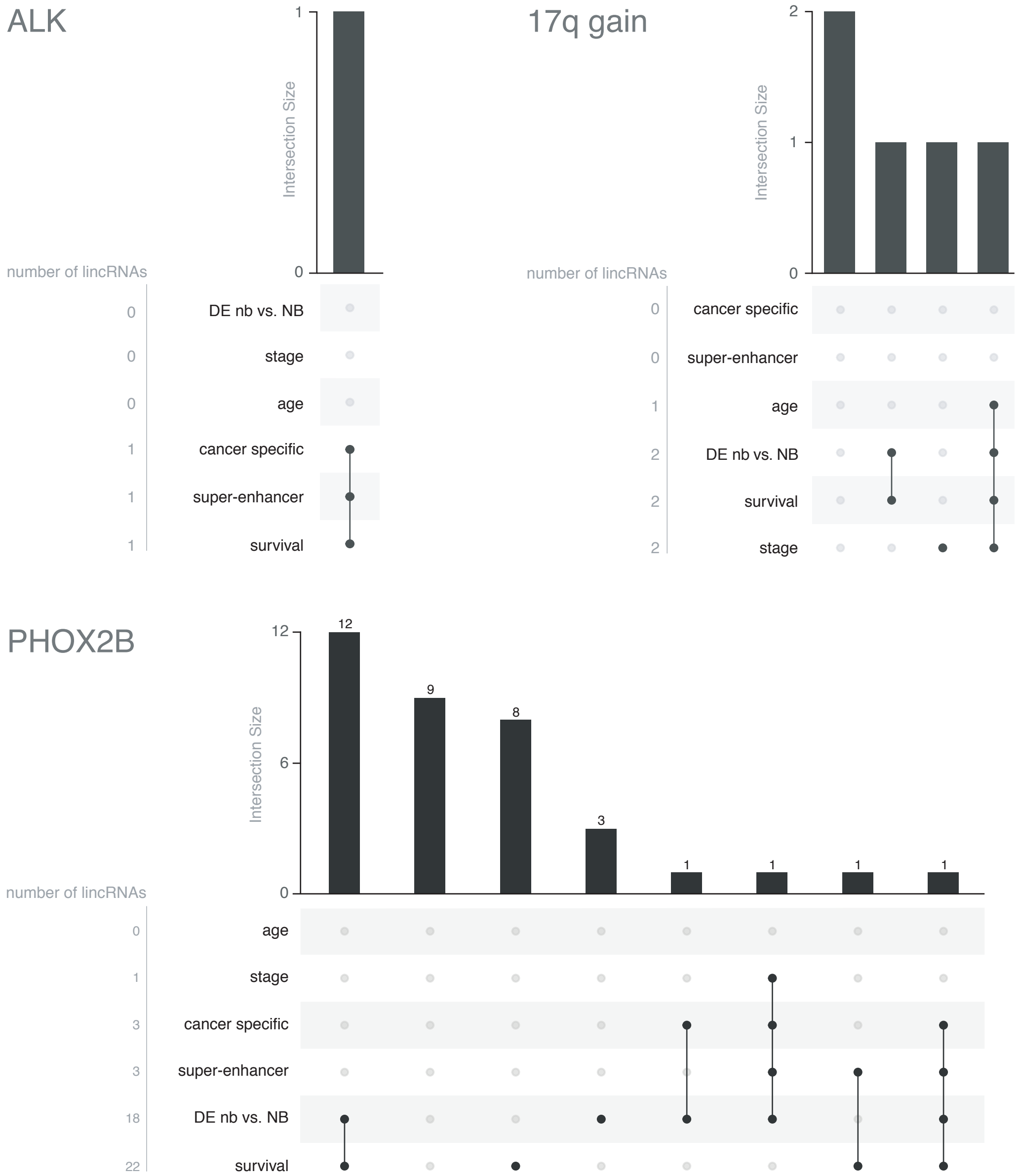

Supplemental Figure 8: Upset plot showing associations with clinical and genetic parameters for lincRNAs found to be regulated by *PHOX2B* or *ALK*, or influenced by a copy number gain of 17q.

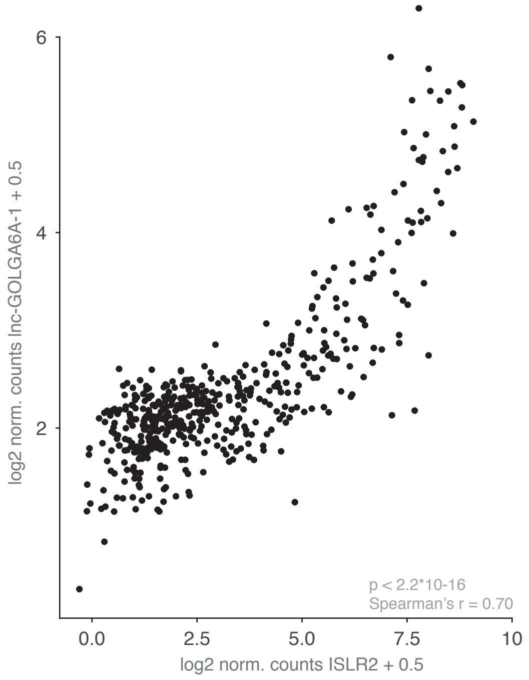

Supplemental Figure 9: Correlation plot of *lnc-GOLGA6A-1* and *ISLR2* expression. Calculation of the correlation coefficient and statistical significance was done using Spearman's method.

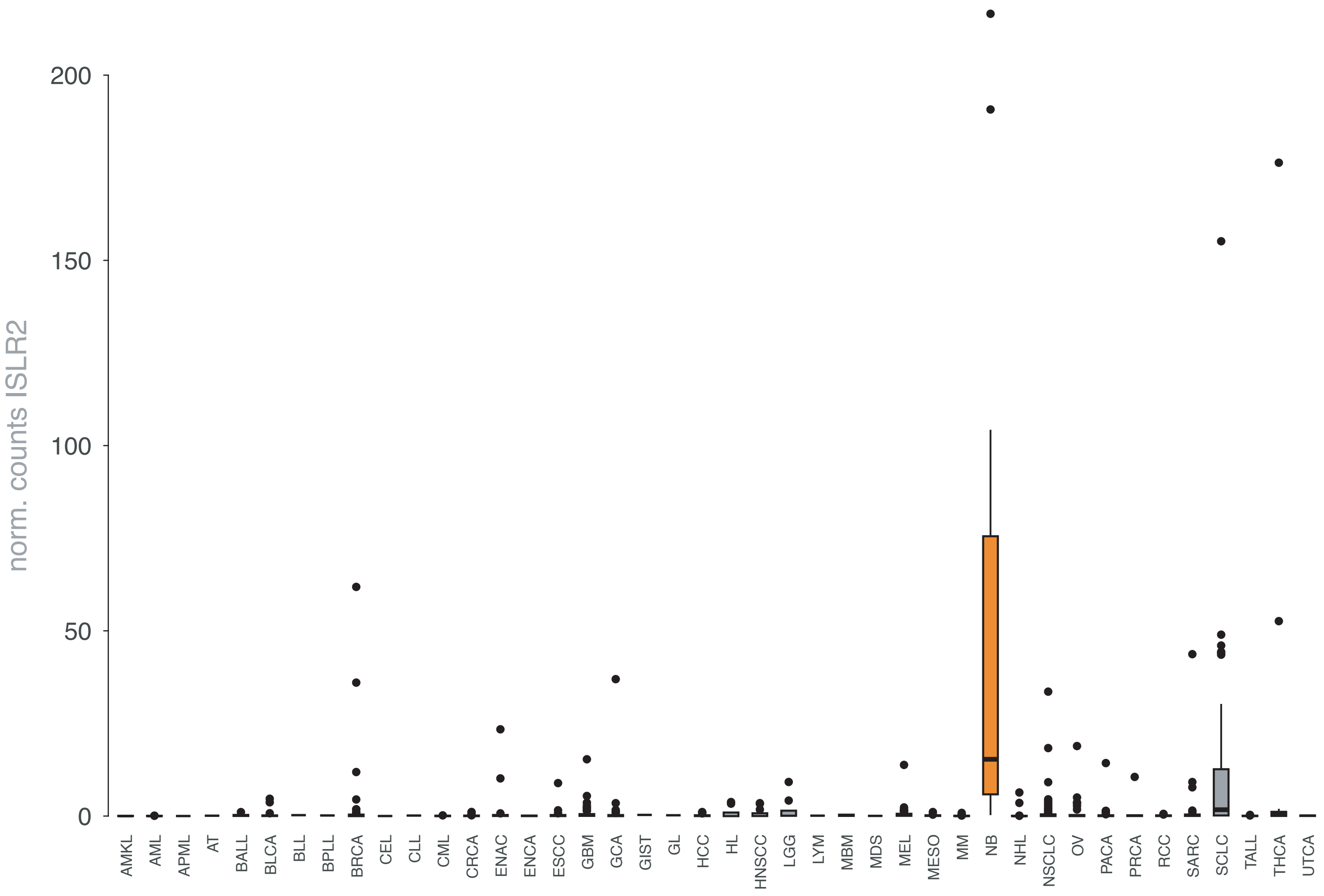

Supplemental Figure 10: Boxplot showing expression pattern of *ISLR2* across different cancer cell lines.

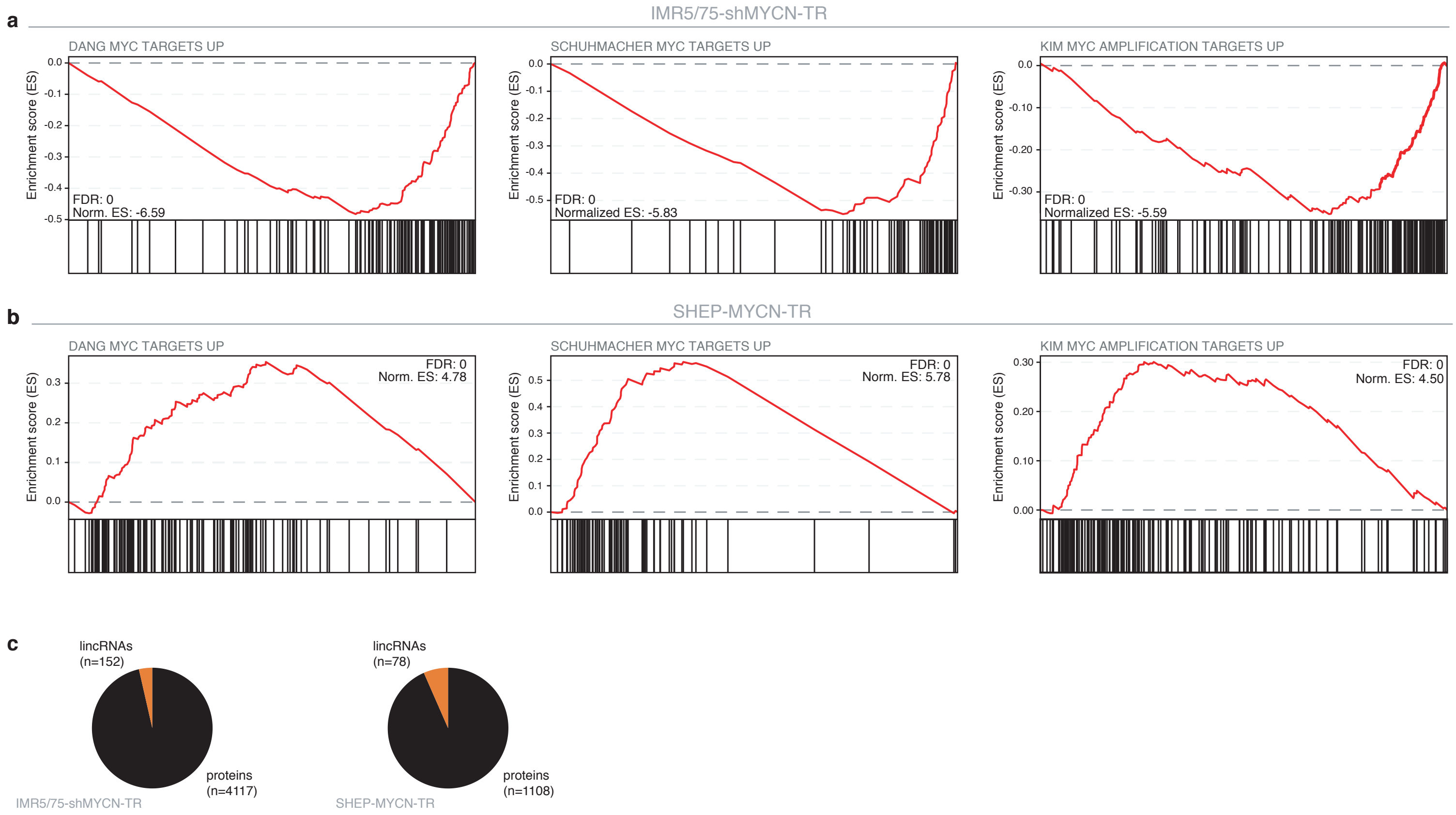

Supplemental Figure 11: a+b) GSEA plots of the *MYCN* model systems, showing for depletion and enrichment for three *MYC* gene sets upon treatment of IMR5/75-shMYCN-TR and SHEP-MYCN-TR, respectively. c) Number of lincRNAs and mRNAs differentially expressed in the modelsystems.

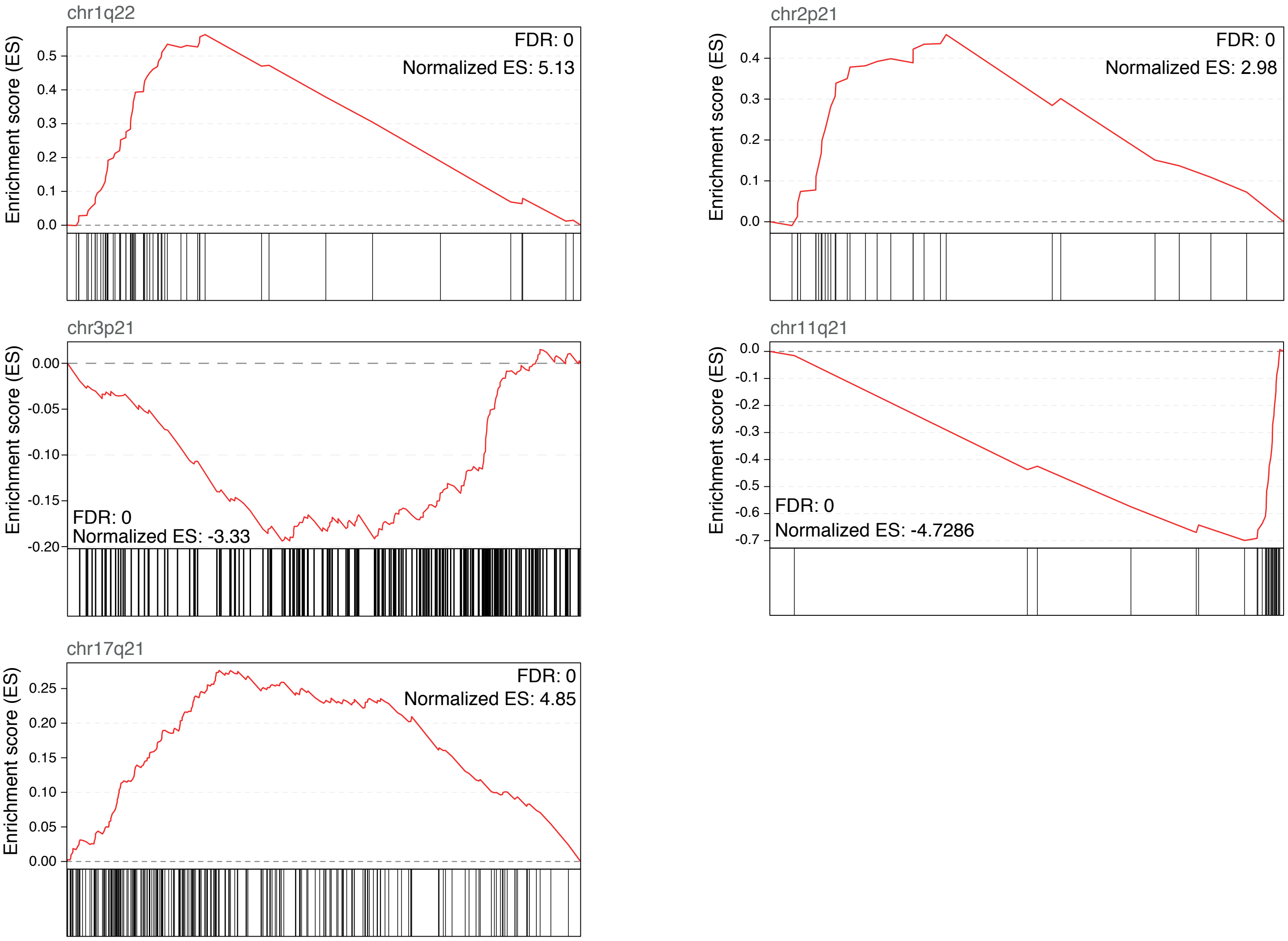

Supplemental Figure 12: GSEA plots showing enrichment/depletion for chromosomal arm gene sets.

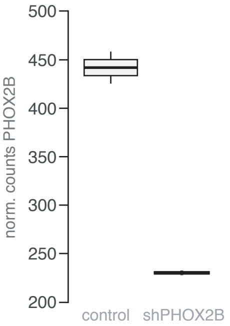

Supplemental Figure 13: Boxplot showing expression levels of *PHOX2B* in samples treated with doxycycline (shPHOX2B) or without doxycycline (control) of the CLB-GA cell line.

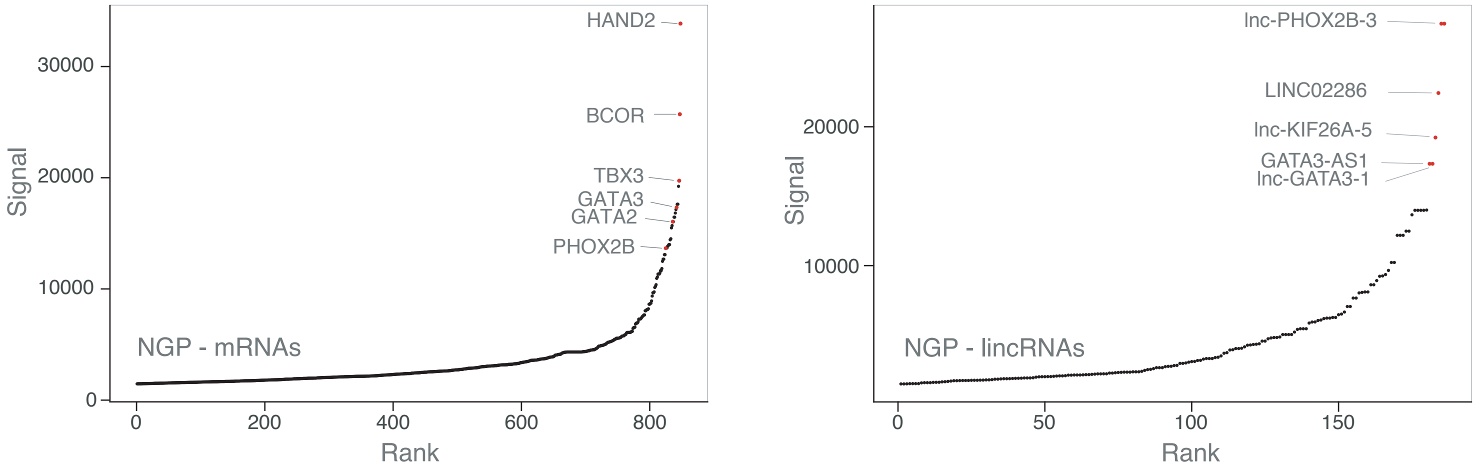

Supplemental Figure 14: Hockeystick plot of genes associated with super-enhancers in NGP for mRNAs and lincRNAs respectively.
